# Climatic variables and ecological modelling data for birds, amphibians and reptiles in the Transboundary Biosphere Reserve of Meseta Ibérica (Portugal-Spain)

**DOI:** 10.3897/BDJ.9.e66509

**Published:** 2021-06-24

**Authors:** João C. Campos, Sara Rodrigues, Teresa Freitas, João A. Santos, João P. Honrado, Adrián Regos

**Affiliations:** 1 InBIO/CIBIO - Centro de Investigação em Biodiversidade e Recursos Genéticos, Campus Agrário de Vairão, Rua Padre Armando Quintas, n° 7, 4485-661 Vairão, Portugal., Porto, Portugal InBIO/CIBIO - Centro de Investigação em Biodiversidade e Recursos Genéticos, Campus Agrário de Vairão, Rua Padre Armando Quintas, n° 7, 4485-661 Vairão, Portugal. Porto Portugal; 2 CITAB - Centro de Investigação e de Tecnologias Agro-Ambientais e Biológicas, Universidade de Trás-os-Montes e Alto Douro, Apartado 1013, 5001-801, Portugal., Vila Real, Portugal CITAB - Centro de Investigação e de Tecnologias Agro-Ambientais e Biológicas, Universidade de Trás-os-Montes e Alto Douro, Apartado 1013, 5001-801, Portugal. Vila Real Portugal; 3 Departamento de Zooloxía, Xenética e Antropoloxía Física, Universidade de Santiago de Compostela, 15782 Santiago de Compostela, Spain., Santiago de Compostela, Spain Departamento de Zooloxía, Xenética e Antropoloxía Física, Universidade de Santiago de Compostela, 15782 Santiago de Compostela, Spain. Santiago de Compostela Spain

**Keywords:** biodiversity, climate change, climate models, conservation, Iberian Peninsula, species distribution models.

## Abstract

**Background:**

Climate change has been widely accepted as one of the major threats for global biodiversity and understanding its potential effects on species distribution is crucial to optimise conservation planning in future scenarios under global change. Integrating detailed climatic data across spatial and temporal scales into species distribution modelling can help to predict potential changes in biodiversity. Consequently, this type of data can be useful for developing efficient biodiversity management and conservation planning. The provision of such data becomes even more important in highly biodiverse regions, currently suffering from climatic and landscape changes. The Transboundary Biosphere Reserve of Meseta Ibérica (BRMI; Portugal-Spain) is one of the most relevant reserves for wildlife in Europe. This highly diverse region is of great ecological and socio-economical interest, suffering from synergistic processes of rural land abandonment and climatic instabilities that currently threaten local biodiversity.

Aiming to optimise conservation planning in the Reserve, we provide a complete dataset of historical and future climate models (1 x 1 km) for the BRMI, used to build a series of distribution models for 207 vertebrate species. These models are projected for 2050 under two climate change scenarios. The climatic suitability of 52% and 57% of the species are predicted to decrease under the intermediate and extreme climatic scenarios, respectively. These models constitute framework data for improving local conservation planning in the Reserve, which should be further supported by implementing climate and land-use change factors to increase the accuracy of future predictions of species distributions in the study area.

**New information:**

Herein, we provide a complete dataset of state-of-the-art historical and future climate model simulations, generated by global-regional climate model chains, with climatic variables resolved at a high spatial resolution (1 × 1 km) over the Transboundary Biosphere Reserve of Meseta Ibérica. Additionally, a complete series of distribution models for 207 species (168 birds, 24 reptiles and 15 amphibians) under future (2050) climate change scenarios is delivered, which constitute framework data for improving local conservation planning in the reserve.

## Introduction

Understanding how species are globally distributed and identifying the key factors that influence their spatial and temporal distribution patterns are essential first steps for solid biodiversity conservation planning (Whittaker et al. 2005). Species distributions are primarily shaped by historical and contemporary events, in which environmental and landscape factors play a decisive role in determining spatial and temporal distribution status and trends (Nogués-Bravo et al. 2018). In this regard, climate change has been widely acknowledged as one of the major current and future threats for global biodiversity (Sippel et al. 2020, Raven and Wagner 2021), causing geographical distribution shifts of a large number of species and, consequently, leading to species extinction events, the disruption of entire ecosystems and also deprivation of human well-being (Pecl et al. 2017, Turner et al. 2020). As such, providing detailed and informative climatic data at both spatial and temporal scales is paramount for better predicting potential environmental impacts on biodiversity and associated ecosystems, which ultimately support optimised conservation planning under global change (Newbold 2018).

One of the most important tools for assisting efficient management and biodiversity conservation planning is species distribution modelling (SDMs; Araújo et al. 2019). These methods derive statistical relationships between geographical species occurrences and environmental predictors (such as climatic factors), which can be consequently used to spatially and temporally predict species distributions under different environmental scenarios (Guisan et al. 2017). In order to efficiently support biodiversity conservation under future environmental conditions, the combined effect of landscape, concrete land cover information and climate factors must be taken into account to improve the model predictive accuracy of potential future changes of species distributions (Triviño et al. 2018, Pausas and Millán 2019).

Improving the predictive power of SDMs becomes paramount in highly biodiverse regions currently under severe climatic and landscape changes. In Europe, Mediterranean rural areas are perfect examples of highly diverse regions from an ecological and socio-economical point of view, suffering from increased effects of landscape and climatic changes (Navarro and Pereira 2012). For instance, the Transboundary Biosphere Reserve of Meseta Ibérica (BRMI), one of the largest reserves and important areas for wildlife in Europe, with around 1,132,000 hectares (www.unesco.org), is currently subjected to processes of rural land abandonment and climatic instabilities that have contributed to the disruption of ecosystem processes (e.g. escalation of extreme wildfires; Sil et al. 2019). The Reserve encompasses five natural parks and several Natura 2000 sites, comprising high landscape heterogeneity and biodiversity. As an example, the Reserve supports a large number of vertebrate species (around 250 species; www.unesco.org), including several emblematic taxa of conservation concern, such as the black stork [*Ciconia nigra* (Linnaeus, 1758)], the Egyptian vulture [*Neophron pernocterus* (Linnaeus, 1766)], the Iberian frog [*Rana iberica* (Boulenger, 1879)] and the Seoane’s viper [*Viper seoanei* (Lataste, 1879)]. However, the current climatic and landscapes changes constitute major threats for the local biodiversity and compiling framework data about how these impacts might influence species distribution patterns in the future could contribute to regional and local conservation efforts.

Here, we present a complete dataset of historical (serving as temporal baseline data) and future climate models with a high spatial resolution (1 × 1 km) for the Transboundary Biosphere Reserve of Meseta Ibérica (Portugal-Spain), as well as a complete series of distribution models for 207 vertebrate species (168 birds, 24 reptiles and 15 amphibians), projected for a historical period (1989-2005) and for future climate change scenarios (2021-2050) in the Reserve.

## General description

### Purpose

These datasets were developed to provide framework data for biodiversity conservation in one of the most diverse Biosphere Reserves in Europe.

### Additional information

**Figure 1.**
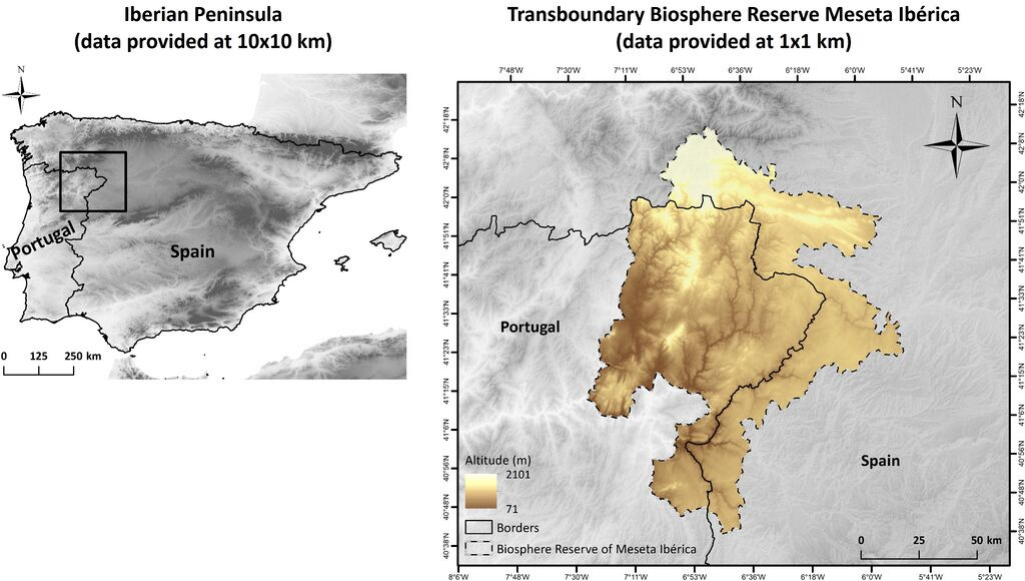
Geographic location of the study areas: the Iberian Peninsula (climate variables and biodiversity data provided at 10 × 10 km resolution) and the Transboundary Biosphere Reserve of Meseta Ibérica (data provided at 1 × 1 km resolution).

The climate model datasets (comprising three main variables – daily total precipitation, maximum and minimum temperatures) are provided for two main areas: the Iberian Peninsula and the Transboundary Biosphere Reserve of Meseta Ibérica (Fig. 1). The climate model simulations are provided for one historical period (daily data from 1989 to 2005) in the Iberian Peninsula (at 9 × 9 km) and two periods (daily data from 1989 to 2005 and from 2021 to 2050) in the Meseta Ibérica (at 1 × 1 km). Future climate data are available from four Global-Regional Climate Model chains and two Representative Concentration Pathways (RCP 4.5 and 8.5). The SDMs are provided for both areas (10 × 10 km in the Iberian Peninsula and 1 × 1 km in the Meseta Ibérica) and for one historical period in the Iberian Peninsula (mean between 1989-2005) and two periods in the Meseta Ibérica (mean between 1989-2005 and mean between 2021 and 2050).

The data are provided in compressed folders, containing the following information:

1. Climate model files encompassing three climatic variables in netCDF format (files organised according to each area and temporal period) and the corresponding bioclimatic variables available in .tiff format;
2. Species models for 207 vertebrate species, including the corresponding spatial projections for the historic and future scenarios (files organised according to each species, area and temporal period).

## Sampling methods

### Step description

Presence/absence data for bird species present in the Iberian Peninsula were obtained from the Spanish and Portuguese Atlas of Breeding Birds, at 10 km resolution (Martí and Del Moral 2003, Equipa Atlas 2008). Presence/absence data for reptile and amphibian species were extracted from the Atlas of Amphibians and Reptiles of Portugal and Spain, at 10 km resolution (Pleguezuelos et al. 2002, Loureiro et al. 2008). Only native species with at least one presence in the BRMI were selected. In addition, species with less than 30 presences in the Iberian Peninsula were excluded to avoid model overfitting (see Araújo et al. 2019). In the end, data were obtained for 207 species: 168 birds, 24 reptiles and 15 amphibians (see Table 1). Taking into account the taxonomic uncertainties of some species (see Table 1), the species list was determined according to the most recently updated versions of the Altases to avoid any taxonomic conflicts (Sillero et al. 2014).

The daily climatic data of temperature and precipitation were retrieved from the E-OBS database v.20.0e (Cornes et al. 2018), from 1989 to 2005. Future climatic data were developed from the following model chains in order to account for potential stochasticity of climate model projections: CNRM-CERFACS-CNRM-CM5 (CNRM), ICHEC-EC-EARTH (ICHEC), IPSL-IPSL-CM5A-MR (IPSL) and MPI-M-MPI-ESM-LR (MPI) models, generated within the EURO-CORDEX project (Jacob et al. 2020) and is available for two Representative Concentration Pathways, one intermediate scenario where emissions start to decline after 2040 (RCP 4.5) and one extreme scenario where emissions experience a continuous increase (RCP 8.5). Climate model data were bias-corrected using quantile mapping and E-OBS as a baseline for the overlapping period between EURO-CORDEX and E-OBS (1989-2005). Both historical and future climate datasets contain three variables: daily total precipitation, maximum and minimum temperatures. For the data collected, temporal and spatial (Biosphere Reserve of Meseta Ibérica and the Iberian Peninsula) domains were extracted and data were bilinearly interpolated to common 9 km grids. Subsequently, a spatial downscaling of temperatures was performed, using the digital elevation model from the Shuttle Radar Topography Mission (SRTM) databases, at 1 km grid resolution and the vertical temperature gradient (altitudinal correction). Precipitation totals were bilinearly interpolated to the same 1 km grid.

The main climate variables (i.e. daily precipitation, maximum temperature and minimum temperature) were used to calculate 19 bioclimatic variables through the “dismo” package from the R software v.4.0.5 (https://www.r-project.org). A Variance Inflation Factor (VIF) analysis between the bioclimatic variables and Spearman correlation tests were conducted using the “usdm” package of R software v.4.0.5 (Suppl. material 1). Highly correlated variables (VIF > 3 and Spearman correlation > 0.7 or < -0.7) were excluded to avoid multicollinearity issues (Guisan et al. 2017). Eight bioclimatic predictors were ultimately selected and implemented in the species distribution models (SDMs; Table 2).

Single-species ensemble models were built for each species at the Iberian Peninsula scale using the “biomod2” R package (Thuiller et al. 2009; http://r-forge.r-project.org/R/?group_id=302) at 10 km resolution. Although the original climate data were obtained at 9 x 9 km, the SDMs were performed at 10 x 10 km to match the spatial resolution of the Atlases' data. Then, the modelling of the climate suitability (hereafter “climate species models”) for each species using the aforementioned bioclimatic variables for 2005 (derived from the mean between 1989 and 2005) was conducted. The ensemble models were built using six modelling techniques (specifically, Generalised Linear Models, Generalised Addictive Models, Random Forests, Artificial Neural Networks, Gradient Boosting Models and Multiple Adaptive Regression Splines), in order to deal with inter-model variabilities (Thuiller et al. 2009). A repeated (10 times) split-sample approach was used to allow independency between model calibration and model evaluation. Each model was trained using 80% of the data, while the remaining 20% were used for model validation using the area under the curve (AUC) of a Receiver-Operating Characteristic (ROC) curve and the True Skill Statistics (TSS). An ensemble-forecasting framework was then applied by stacking the single-species models using a weighted average approach available in “biomod2”, using AUC values as model weights.

**Figure 2.**
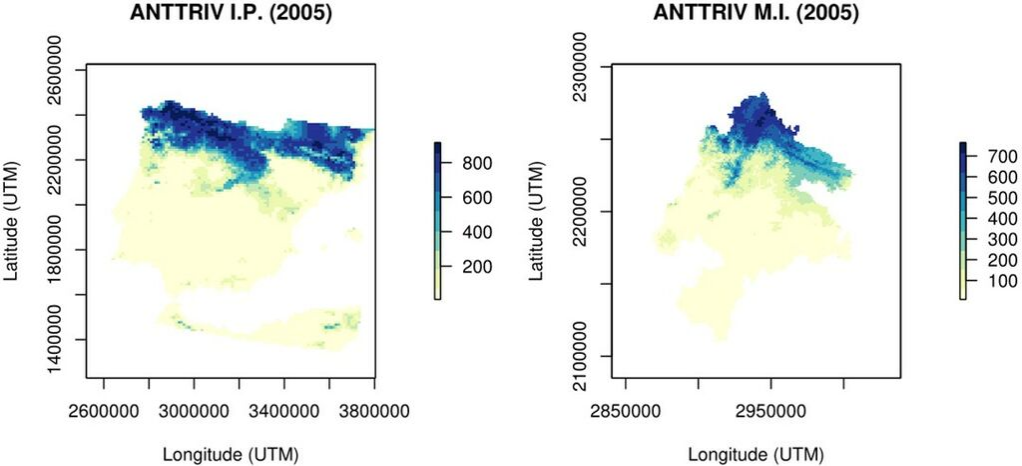
Example of the historical climate (1989-2005) model projections obtained for the Iberian Peninsula (I.P.; 10 × 10 km) and the Transboundary Biosphere Reserve of Meseta Ibérica (M.I.; 1 × 1 km). The models present the ensemble suitability values for the Tree pipit (*Anthus trivialis*; code: ANTRRIV).

**Figure 3.**
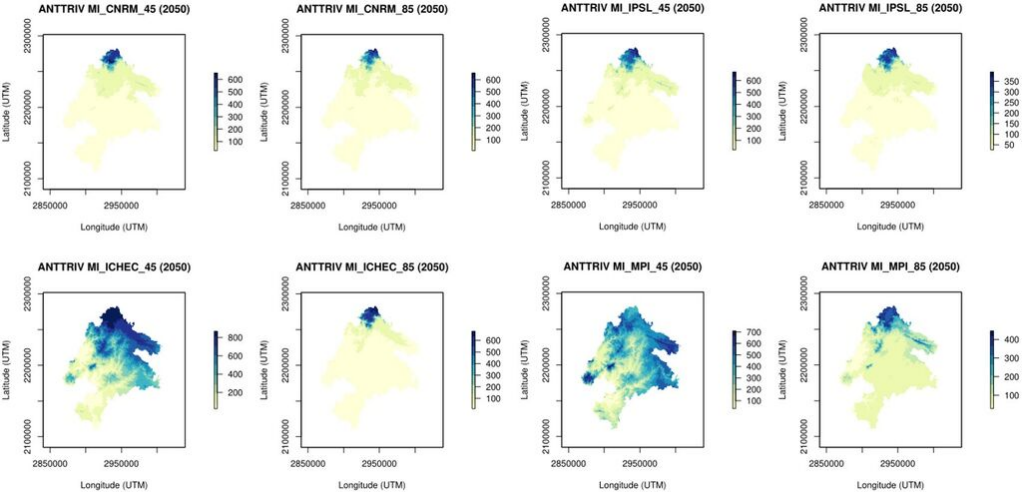
Example of future climate model projections for 2050 obtained for the Transboundary Biosphere Reserve of Meseta Ibérica (M.I.; 1 × 1 km). The models present the ensemble suitability values for the Tree pipit (*Anthus trivialis*; code: ANTRRIV), according to each climate model (CNRM, IPSL, ICHEC and MPI; Jacob et al. 2020) and each Representative Concentration Pathways scenarios (RCP 4.5; RCP 8.5).

The ensemble models were then projected to the Meseta Ibérica at 1 km resolution for the historical (1989-2005; Fig. 2) and future (2021-2050) periods for the four climate models and two RCP scenarios (Fig. 3). Finally, ensemble model predictions were reclassified into binary presence/absence maps through ROC optimised thresholds available in the “biomod2” package (see Thuiller et al. 2009).

This dataset contributes towards updating the current knowledge on the potential effects of climate change on the distribution of three main taxonomic groups in one of the largest Biosphere Reserves in Europe. In general, a wide range of species responses to climate change were observed, which might be explained by species-specific ecological preferences. The extent of species responses varied according to the four climate models due to the potential stochasticity of climate projections, but the predicted positive or negative climatic effects were congruent amongst all models for each species (see Fig. 3). According to the SDMs, the majority of species are expected to be negatively affected by climate change scenarios (see Fig. 3). In fact, climatic suitable areas for 52% and 57% of the species are predicted to decrease under the intermediate (RCP 4.5) and extreme (RCP 8.5) climate change scenarios, respectively (see example in Fig. 3). Future climatic instabilities might contribute to distribution contractions and shifts, which might increase species vulnerability to extinction due to stochastic effects. Nonetheless, future studies should focus on combining the effects of land-use change and climate factors, in order to improve model predictive accuracy of future impacts on species distributions and, thus, to better support conservation planning and actions in the study area.

## Geographic coverage

### Description

The geographic range of the data covers the entire continental area of the Iberian Peninsula at 10 km of spatial resolution (45.158ºN and 35.347ºN Latitude; 9.560ºW and 3.889ºE Longitude) and the Transboundary Biosphere Reserve of Meseta Ibérica at 1 km of spatial resolution (42.384ºN and 40.588ºN Latitude; 7.692ºW and 5.613ºW Longitude).

### Coordinates

40.588 and 42.384 Latitude; -7.692 and -5.613 Longitude.

## Temporal coverage

### Notes

Climate data cover the historical period between 1989 and 2005 (daily data) and a future period between 2020 and 2050 (daily data of four climate models under the RCP 4.5 and RCP 8.5 scenarios).

Species distribution models (climate species models) for the 207 vertebrate species cover the historical period of 2005 (average of the bioclimatic variables between 1989 and 2005) and a future period of 2050 (average between 2020 and 2050, for each of the four climate models and RCP scenarios).

## Usage licence

### Usage licence

Creative Commons Public Domain Waiver (CC-Zero)

## Data resources

### Data package title

Climate models and species distribution models of amphibians, birds and reptiles of the Iberian peninsula and the Biosphere Reserve of Meseta Ibérica)

### Number of data sets

2

### Data set 1.

#### Data set name

Climate models

#### Data format

netCDF (.nc)

#### Number of columns

2

#### Download URL

Part1: https://zenodo.org/record/4589376#.YFTl3dxUnIU Part 2: https://zenodo.org/record/4590027#.YFTmBdxUnIU

#### Description

Daily climate variables (daily precipitation, maximum temperature and minimum temperature) for a historical (1989-2005) and future period (2021-2050), for four climate models (CNRM, ICHEC, IPSL and MPI) and two Representative Concentration Pathways (RCP 4.5 and 8.5). Climatic variables are provided at 9 × 9 km resolution for the Iberian Peninsula (only for the historical period) and at 1 × 1 km and for the Transboundary Biosphere Reserve of Meseta Ibérica (both periods). Data divided into two parts.

**Data set 1.** 

Column label	Column description
Files of the historic period - AREA_EOBS_H_ALT_VAR_1	Code description - AREA refers to the Iberian Peninsula (PI) or Meseta Ibérica (MI), EOBS to the historic climatic dataset of reference (E-OBS), H to the historical period (H), ALT to the altitudinal-based correction of climate variables, VAR to the three provided variables (RR - daily preciptation; TMAX - Maximum temperature; TMIN - Minimum temperature) and 1 to the spatial resolution (1 km).
Files of the future period - MI_MODEL_RCP_MR_ALT_VAR_1	Code description - MI refers to the Meseta Ibérica, MODEL to the climate model used (CNRM-CERFACS-CNRM-CM5 - CNRM; ICHEC-EC-EARTH - ICHEC; IPSL-IPSL-CM5A-MR - IPSL; MPI-M-MPI-ESM-LR - MPI), RCP to the Representative Concentration Pathway (RCP 4.5 - 45; RCP 8.5 - 85), MR to the future period, ALT to the altitudinal-based correction of climate variables, VAR to the three provided variables (RR - daily preciptation; TMAX - Maximum temperature; TMIN - Minimum temperature) and 1 to the spatial resolution (1 km).

### Data set 2.

#### Data set name

Species distribution models

#### Number of columns

1

#### Download URL

Part 1: https://zenodo.org/record/4598254#.YFTkjdxUnIU Part 2: https://zenodo.org/record/4599822#.YFTlv9xUnIU

#### Description

Species distribution models of 207 vertebrates distributed in the Iberian Peninsula and the Transboundary Biosphere Reserve of Meseta Ibérica. The models are available at 10 × 10 km resolution for the Iberian Peninsula (climate models for 2005). Model projections are available for 2005 and 2050 (for the CNRM, ICHEC, IPSL and MPI climate models and the RCP 4.5 and RCP 8.5 scenarios) for the Biosphere Reserve at 1 × 1 km resolution. Data divided into two parts.

**Data set 2.** 

Column label	Column description
Climate models	Species distribution models of 207 vertebrates for 2005 and 2050

## Supplementary Material

7C054ED9-E679-5200-B125-2F31598F657B 10.3897/BDJ.9.e66509.suppl1 Supplementary material 1 Pearson correlation analysis between bioclimatic variables Data type Statistical analyses File: oo_524471.pdf https://binary.pensoft.net/file/524471 João C. Campos; Sara Rodrigues; Teresa Freitas; João A. Santos; João P. Honrado, Adrián Regos

## Figures and Tables

**Table 1.** Species information: taxonomic group, scientific name, species code and number of presences used for modelling (N). The quality threshold (area under the curve - AUC) used for model selection (to be included on ensemble modelling) are indicated. The accuracy metrics of ensemble species distribution models (SDMs), measured by the AUC and True Skill Statistics (TSS), are also mentioned. Ten model replicates were conducted for each species.

**Group**	**Scientific name**	**Code**	**N**	**AUC threshold**	**Climate models**	
					**AUC**	**TSS**
** Amphibia **	*Alytes cisternasii*	ACI	1253	0.8	0.96	0.795
** Amphibia **	*Alytes obstetricans*	AOB	2336	0.8	0.927	0.681
** Amphibia **	*Bufo spinosus*	BSP	4471	0.7	0.915	0.654
** Amphibia **	*Discoglossus galganoi*	DGA	1930	0.7	0.993	0.924
** Amphibia **	*Epidalea calamita*	ECA	3973	0.7	0.949	0.757
** Amphibia **	*Hyla molleri*	HMO	1502	0.8	0.957	0.759
** Amphibia **	*Lissotriton boscai*	LBO	1695	0.8	0.948	0.76
** Amphibia **	*Lissotriton helveticus*	LHE	701	0.8	0.971	0.833
** Amphibia **	*Pelobates cultripes*	PCU	2221	0.8	0.968	0.786
** Amphibia **	*Pelophylax perezi*	PPE	5587	0.8	0.989	0.932
** Amphibia **	*Pelodytes punctatus*	PPU	1765	0.7	0.95	0.776
** Amphibia **	*Pleurodeles waltl*	PWA	1897	0.8	0.918	0.659
** Amphibia **	*Rana iberica*	RIB	953	0.8	0.984	0.871
** Amphibia **	*Salamandra salamandra* spp.	SSA	2422	0.8	0.928	0.706
** Amphibia **	*Triturus marmoratus* spp.	TMA	2485	0.7	0.924	0.673
**Birds**	*Accipiter gentilis*	ACCGENT	2266	0.7	0.991	0.895
**Birds**	*Accipiter nisus*	ACCNISU	2565	0.7	0.984	0.88
**Birds**	*Acrocephalus arundinaceus*	ACRARUN	1348	0.8	0.99	0.908
**Birds**	*Acrocephalus scirpaceus*	ACRSCIR	1581	0.7	0.991	0.912
**Birds**	*Aegithalos caudatus*	AEGCAUD	4157	0.7	0.888	0.599
**Birds**	*Alauda arvensis*	ALAARVE	2999	0.8	0.896	0.62
**Birds**	*Alcedo atthis*	ALCATTH	2285	0.7	0.861	0.542
**Birds**	*Alectoris rufa*	ALERUFA	5050	0.7	0.946	0.803
**Birds**	*Anas clypeata*	ANACLYP	141	0.8	0.987	0.945
**Birds**	*Anas platyrhynchos*	ANAPLAT	3354	0.7	0.871	0.56
**Birds**	*Anas strepera*	ANASTRE	305	0.8	0.981	0.913
**Birds**	*Anthus campestris*	ANTCAMP	2248	0.8	0.896	0.614
**Birds**	*Anthus spinoletta*	ANTSPIN	439	0.8	0.987	0.908
**Birds**	*Anthus trivialis*	ANTTRIV	1163	0.8	0.97	0.846
**Birds**	*Apus melba*	APUMELB	1047	0.7	0.975	0.849
**Birds**	*Apus pallidus*	APUPALL	847	0.8	0.945	0.75
**Birds**	*Aquila chrysaetos*	AQUCHRY	700	0.7	0.968	0.835
**Birds**	*Ardea cinerea*	ARDCINE	543	0.7	0.994	0.944
**Birds**	*Ardea purpurea*	ARDPURP	259	0.8	0.977	0.872
**Birds**	*Asio flammeus*	ASIFLAM	77	0.8	0.991	0.973
**Birds**	*Asio otus*	ASIOTUS	1362	0.7	0.893	0.597
**Birds**	*Athene noctua*	ATHNOCT	4424	0.7	0.962	0.793
**Birds**	*Aythya ferina*	AYTFERI	195	0.8	0.987	0.94
**Birds**	*Bubo bubo*	BUBBUBO	2141	0.7	0.88	0.601
**Birds**	*Bubulcus ibis*	BUBIBIS	287	0.8	0.964	0.827
**Birds**	*Burhinus oedicnemus*	BUROEDI	2264	0.8	0.975	0.836
**Birds**	*Buteo buteo*	BUTBUTE	4504	0.7	0.867	0.546
**Birds**	*Calandrella brachydactyla*	CALBRAC	2245	0.8	0.992	0.909
**Birds**	*Alauda rufescens*	CALRUFE	246	0.8	0.985	0.903
**Birds**	*Caprimulgus europaeus*	CAPEURO	1979	0.8	0.899	0.618
**Birds**	*Caprimulgus ruficollis*	CAPRUFI	1781	0.8	0.916	0.656
**Birds**	*Carduelis spinus*	CARSPIN	84	0.8	0.99	0.963
**Birds**	*Hirundo daurica*	CECDAUR	1253	0.8	0.992	0.952
**Birds**	*Certhia brachydactyla*	CERBRAC	2336	0.7	0.868	0.56
**Birds**	*Cettia cetti*	CETCETT	4471	0.7	0.927	0.674
**Birds**	*Charadrius dubius*	CHADUBI	1930	0.7	0.989	0.896
**Birds**	*Chersophilus duponti*	CHEDUPO	3973	0.8	0.98	0.907
**Birds**	*Chlidonias hybrida*	CHLHYBR	1502	0.8	0.991	0.959
**Birds**	*Ciconia ciconia*	CICCICO	1695	0.8	0.927	0.705
**Birds**	*Ciconia nigra*	CICNIGR	701	0.8	0.964	0.838
**Birds**	*Cinclus cinclus*	CINCINC	2221	0.8	0.937	0.728
**Birds**	*Circus aeruginosus*	CIRAERU	5587	0.8	0.979	0.891
**Birds**	*Circus cyaneus*	CIRCYAN	1765	0.8	0.963	0.832
**Birds**	*Circaetus gallicus*	CIRGALL	1897	0.7	0.944	0.728
**Birds**	*Circus pygargus*	CIRPYGA	953	0.7	0.992	0.913
**Birds**	*Cisticola juncidis*	CISJUNC	2422	0.8	0.97	0.814
**Birds**	*Clamator glandarius*	CLAGLAN	2485	0.7	0.994	0.925
**Birds**	*Coccothraustes coccothraustes*	COCCOCC	2266	0.8	0.965	0.818
**Birds**	*Columba livia*	COLLIVI	2565	0.7	0.945	0.787
**Birds**	*Columba oenas*	COLOENA	1348	0.8	0.917	0.68
**Birds**	*Columba palumbus*	COLPALU	1581	0.7	0.947	0.793
**Birds**	*Corvus corone*	CORCORO	4157	0.8	0.936	0.701
**Birds**	*Coracias garrulus*	CORGARR	2999	0.8	0.927	0.705
**Birds**	*Corvus monedula*	CORMONE	2285	0.7	0.992	0.902
**Birds**	*Coturnix coturnix*	COTCOTU	5050	0.7	0.934	0.717
**Birds**	*Cuculus canorus*	CUCCANO	141	0.7	0.98	0.856
**Birds**	*Cyanopica cyana*	CYACYAN	3354	0.8	0.954	0.765
**Birds**	*Dendrocopos major*	DENMAJO	305	0.8	0.974	0.814
**Birds**	*Dendrocopos minor*	DENMINO	2248	0.8	0.95	0.751
**Birds**	*Egretta garzetta*	EGRGARZ	439	0.8	0.976	0.878
**Birds**	*Elanus caeruleus*	ELACAER	1163	0.8	0.943	0.734
**Birds**	*Emberiza calandra*	EMBCALA	1047	0.7	0.908	0.695
**Birds**	*Emberiza cia*	EMBCIA	847	0.8	0.94	0.681
**Birds**	*Emberiza cirlus*	EMBCIRL	700	0.7	0.991	0.901
**Birds**	*Emberiza citrinella*	EMBCITR	543	0.8	0.983	0.898
**Birds**	*Emberiza hortulana*	EMBHORT	259	0.8	0.947	0.755
**Birds**	*Erithacus rubecula*	ERIRUBE	77	0.8	0.905	0.619
**Birds**	*Falco naumanni*	FALNAUM	1362	0.8	0.93	0.723
**Birds**	*Falco peregrinus*	FALPERE	4424	0.8	0.99	0.892
**Birds**	*Falco subbuteo*	FALSUBB	195	0.7	0.975	0.819
**Birds**	*Ficedula hypoleuca*	FICHYPO	2141	0.8	0.975	0.899
**Birds**	*Fringilla coelebs*	FRICOEL	287	0.7	0.901	0.644
**Birds**	*Fulica atra*	FULATRA	2264	0.8	0.927	0.688
**Birds**	*Gallinula chloropus*	GALCHLO	4504	0.7	0.874	0.593
**Birds**	*Galerida cristata*	GALCRIS	2245	0.8	0.934	0.701
**Birds**	*Galerida theklae*	GALTHEK	246	0.8	0.943	0.710
**Birds**	*Garrulus glandarius*	GARGLAN	1979	0.8	0.945	0.717
**Birds**	*Gyps fulvus*	GYPFULV	1781	0.7	0.999	0.98
**Birds**	*Hieraaetus fasciatus*	HIEFASC	84	0.8	0.997	0.956
**Birds**	*Hieraaetus pennatus*	HIEPENN	1253	0.7	0.99	0.889
**Birds**	*Himantopus himantopus*	HIMHIMA	2336	0.8	0.921	0.668
**Birds**	*Ixobrychus minutus*	IXOMINU	4471	0.8	0.991	0.944
**Birds**	*Jynx torquilla*	JYNTORQ	1930	0.7	0.989	0.891
**Birds**	*Lanius collurio*	LANCOLL	3973	0.8	0.971	0.855
**Birds**	*Lanius excubitor*	LANEXCU	1502	0.7	0.885	0.611
**Birds**	*Lanius senator*	LANSENA	1695	0.8	0.947	0.761
**Birds**	*Larus ridibundus*	LARRIDI	701	0.8	0.994	0.968
**Birds**	*Loxia curvirostra*	LOXCURV	2221	0.8	0.931	0.733
**Birds**	*Lullula arborea*	LULARBO	5587	0.7	0.99	0.897
**Birds**	*Luscinia megarhynchos*	LUSMEGA	1765	0.7	0.992	0.923
**Birds**	*Cyanecula svecica*	LUSSVEC	1897	0.8	0.995	0.969
**Birds**	*Melanocorypha calandra*	MELCALA	953	0.8	0.918	0.681
**Birds**	*Merops apiaster*	MERAPIA	2422	0.8	0.938	0.717
**Birds**	*Milvus migrans*	MILMIGR	2485	0.7	0.976	0.835
**Birds**	*Milvus milvus*	MILMILV	2266	0.8	0.938	0.727
**Birds**	*Monticola saxatilis*	MONSAXA	2565	0.8	0.941	0.751
**Birds**	*Monticola solitarius*	MONSOLI	1348	0.8	0.992	0.908
**Birds**	*Motacilla alba*	MOTALBA	1581	0.7	0.971	0.864
**Birds**	*Motacilla cinerea*	MOTCINE	4157	0.8	0.94	0.7
**Birds**	*Motacilla flava*	MOTFLAV	2999	0.8	0.97	0.836
**Birds**	*Muscicapa striata*	MUSSTRI	2285	0.7	0.977	0.835
**Birds**	*Neophron percnopterus*	NEOPERC	5050	0.7	0.97	0.876
**Birds**	*Nycticorax nycticorax*	NYCNYCT	141	0.8	0.995	0.974
**Birds**	*Oenanthe hispanica*	OENHISP	3354	0.8	0.909	0.686
**Birds**	*Oenanthe leucura*	OENLEUC	305	0.8	0.945	0.754
**Birds**	*Oenanthe oenanthe*	OENOENA	2248	0.8	0.923	0.674
**Birds**	*Oriolus oriolus*	ORIORIO	439	0.7	0.91	0.666
**Birds**	*Otis tarda*	OTITARD	1163	0.8	0.961	0.797
**Birds**	*Otus scops*	OTUSCOP	1047	0.7	0.925	0.695
**Birds**	*Periparus ater*	PARATER	847	0.8	0.92	0.669
**Birds**	*Parus caeruleus*	PARCAER	700	0.7	0.884	0.599
**Birds**	*Parus cristatus*	PARCRIS	543	0.8	0.985	0.863
**Birds**	*Parus major*	PARMAJO	259	0.7	0.935	0.745
**Birds**	*Passer hispaniolensis*	PASHISP	77	0.8	0.942	0.736
**Birds**	*Passer montanus*	PASMONT	1362	0.7	0.869	0.541
**Birds**	*Pernis apivorus*	PERAPIV	4424	0.8	0.937	0.736
**Birds**	*Perdix perdix*	PERPERD	195	0.8	0.993	0.954
**Birds**	*Petronia petronia*	PETPETR	2141	0.8	0.905	0.63
**Birds**	*Phasianus colchicus*	PHACOLC	287	0.8	0.997	0.985
**Birds**	*Phoenicurus ochruros*	PHOOCHR	2264	0.8	0.91	0.632
**Birds**	*Phoenicurus phoenicurus*	PHOPHOE	4504	0.8	0.949	0.77
**Birds**	*Phylloscopus bonelli*	PHYBONE	2245	0.8	0.906	0.626
**Birds**	*Phylloscopus collybita*	PHYCOLL	246	0.8	0.922	0.678
**Birds**	*Phylloscopus ibericus*	PHYIBER	1979	0.8	0.935	0.729
**Birds**	*Pica pica*	PICPICA	1781	0.7	0.86	0.536
**Birds**	*Picus viridis*	PICVIRI	84	0.7	0.868	0.551
**Birds**	*Podiceps cristatus*	PODCRIS	1253	0.8	0.978	0.889
**Birds**	*Podiceps nigricollis*	PODNIGR	2336	0.8	0.993	0.962
**Birds**	*Prunella collaris*	PRUCOLL	4471	0.8	0.994	0.957
**Birds**	*Prunella modularis*	PRUMODU	1930	0.8	0.976	0.844
**Birds**	*Pterocles alchata*	PTEALCH	3973	0.8	0.974	0.877
**Birds**	*Pterocles orientalis*	PTEORIE	1502	0.8	0.968	0.84
**Birds**	*Ptyonoprogne rupestris*	PTYRUPE	1695	0.8	0.992	0.902
**Birds**	*Pyrrhocorax graculus*	PYRGRAC	701	0.8	0.992	0.947
**Birds**	*Pyrrhula pyrrhula*	PYRPYRR	2221	0.8	0.917	0.681
**Birds**	*Rallus aquaticus*	RALAQUA	5587	0.7	0.995	0.948
**Birds**	*Recurvirostra avosetta*	RECAVOS	1765	0.8	0.99	0.945
**Birds**	*Regulus ignicapillus*	REGIGNI	1897	0.8	0.928	0.693
**Birds**	*Regulus regulus*	REGREGU	953	0.8	0.928	0.899
**Birds**	*Remiz pendulinus*	REMPEND	2422	0.8	0.966	0.824
**Birds**	*Riparia riparia*	RIPRIPA	2485	0.7	0.993	0.932
**Birds**	*Saxicola rubetra*	SAXRUBE	2266	0.8	0.978	0.888
**Birds**	*Saxicola torquatus*	SAXTORQ	2565	0.7	0.898	0.622
**Birds**	*Serinus citrinella*	SERCITR	1348	0.8	0.984	0.904
**Birds**	*Sitta europaea*	SITEURO	1581	0.8	0.949	0.736
**Birds**	*Sterna nilotica*	STENILO	4157	0.8	0.996	0.981
**Birds**	*Strix aluco*	STRALUC	2999	0.7	0.991	0.896
**Birds**	*Streptopelia decaocto*	STRDECA	2285	0.7	0.898	0.651
**Birds**	*Streptopelia turtur*	STRTURT	5050	0.7	0.927	0.697
**Birds**	*Sturnus unicolor*	STUUNIC	141	0.7	0.923	0.71
**Birds**	*Sylvia atricapilla*	SYLATRI	3354	0.7	0.991	0.902
**Birds**	*Sylvia borin*	SYLBORI	305	0.8	0.931	0.712
**Birds**	*Sylvia cantillans*	SYLCANT	2248	0.8	0.896	0.602
**Birds**	*Sylvia communis*	SYLCOMM	439	0.7	0.899	0.606
**Birds**	*Sylvia conspicillata*	SYLCONS	1163	0.8	0.947	0.747
**Birds**	*Sylvia hortensis*	SYLHORT	1047	0.7	0.983	0.881
**Birds**	*Sylvia melanocephala*	SYLMELA	847	0.8	0.926	0.663
**Birds**	*Sylvia undata*	SYLUNDA	700	0.7	0.906	0.643
**Birds**	*Tachybaptus ruficollis*	TACRUFI	543	0.7	0.967	0.817
**Birds**	*Tetrax tetrax*	TETTETR	259	0.8	0.988	0.913
**Birds**	*Tichodroma muraria*	TICMURA	77	0.8	0.997	0.975
**Birds**	*Tringa totanus*	TRITOTA	1362	0.8	0.994	0.98
**Birds**	*Troglodytes troglodytes*	TROTROG	4424	0.8	0.931	0.667
**Birds**	*Turdus philomelos*	TURPHIL	195	0.8	0.936	0.704
**Birds**	*Turdus viscivorus*	TURVISC	2141	0.7	0.896	0.637
**Birds**	*Tyto alba*	TYTALBA	287	0.7	0.947	0.749
**Birds**	*Upupa epops*	UPUEPOP	2264	0.7	0.904	0.66
**Birds**	*Vanellus vanellus*	VANVANE	4504	0.8	0.979	0.927
** Reptilia **	*Acanthodactylus erythrurus*	AER	2245	0.7	0.932	0.73
** Reptilia **	*Anguis fragilis*	AFR	246	0.8	0.957	0.781
** Reptilia **	*Blanus cinereus*	BCI	1979	0.8	0.914	0.655
** Reptilia **	*Coronella austriaca*	CAU	1781	0.8	0.954	0.787
** Reptilia **	*Chalcides bedriagai*	CBE	84	0.7	0.993	0.943
** Reptilia **	*Coronella girondica*	CGI	1253	0.7	0.932	0.715
** Reptilia **	*Chalcides striatus*	CST	2336	0.7	0.993	0.924
** Reptilia **	*Emys orbicularis* spp.	EOR	4471	0.8	0.996	0.954
** Reptilia **	*Hemorrhois hippocrepis*	HHI	1930	0.8	0.918	0.692
** Reptilia **	*Iberolacerta monticola* spp.	IMO	3973	0.8	0.995	0.965
** Reptilia **	*Lacerta schreiberi*	LSC	1502	0.8	0.971	0.831
** Reptilia **	*Macroprotodon brevis* spp.	MBR	1695	0.8	0.943	0.732
** Reptilia **	*Mauremys leprosa*	MLE	701	0.8	0.918	0.661
** Reptilia **	*Malpolon monspessulanus*	MMO	2221	0.7	0.973	0.868
** Reptilia **	*Natrix astreptophora*	NAS	5587	0.7	0.866	0.543
** Reptilia **	*Natrix maura*	NMA	1765	0.7	0.966	0.809
** Reptilia **	*Psammodromus algirus*	PAL	1897	0.8	0.916	0.677
** Reptilia **	*Podarcis bocagei*	PBO	953	0.8	0.994	0.95
** Reptilia **	*Podarcis guadarramae*	PGU	2422	0.7	0.984	0.885
** Reptilia **	*Timon lepidus* spp.	TLE	2485	0.7	0.944	0.746
** Reptilia **	*Tarentola mauritanica*	TMR	2266	0.8	0.914	0.674
** Reptilia **	*Vipera latastei*	VLA	2565	0.7	0.994	0.931
** Reptilia **	*Vipera seoanei*	VSE	1348	0.8	0.986	0.93
** Reptilia **	*Zamenis scalaris*	ZSC	1581	0.7	0.866	0.574

**Table 2.** Description of the bioclimatic variables used in species distribution models. The code, name, units and the regional (Iberian Peninsula) and local (Biosphere Reserve of Meseta Ibérica) ranges are indicated for each variable.

**Code**	**Variable name**	**Units**	**Iberian Peninsula**	**Meseta Ibérica**
BIO3	Isothermality	Coefficient	25 – 43	33 - 40
BIO4	Temperature Seasonality	Coefficient	387 - 870	666 - 813
BIO10	Mean Temperature of Warmest Quarter	ºC	11.2 – 28.4	15.2 – 26.8
BIO11	Mean Temperature of Coldest Quarter	ºC	-7.8 – 12.9	-3.1 – 6.7
BIO15	Precipitation Seasonality	Coefficient	23 – 94	47 - 76
BIO16	Precipitation of Wettest Quarter	mm	200 - 2200	510 - 1110
BIO17	Precipitation of Driest Quarter	mm	0 - 470	0 - 130
BIO19	Precipitation of Coldest Quarter	mm	30 - 1130	120 - 470
