# Supplementary material for: Climatic variables and ecological modelling data for birds, amphibians and reptiles in the Transboundary Biosphere Reserve of Meseta Ibérica (Portugal-Spain)
Source: Biodivers Data J. 2021 Jun 24;9:e66509. doi: 10.3897/BDJ.9.e66509 (PMC8249360; doi:10.3897/BDJ.9.e66509)
Supplement: Supplementary material 1 — Pearson correlation analysis between bioclimatic variables [file bdj-09-e66509-s001.pdf]

Table S1. Matrix indicating the results of the Pearson correlation analysis between bioclimatic variables. Codes refer to: BIO1 - Annual Mean Temperature; BIO3 - Isothermality; BIO4 - Temperature Seasonality; BIO5 - Max Temperature of Warmest Month; BIO6 - Min Temperature of Coldest Month; BIO7 - Temperature Annual Range; BIO10 - Mean Temperature of Warmest Quarter; BIO11 - Mean Temperature of Coldest Quarter; BIO12 - Annual Precipitation; BIO13 - Precipitation of Wettest Month; BIO14 - Precipitation of Driest Month; BIO15 - Precipitation Seasonality; BIO16 - Precipitation of Wettest Quarter; BIO17 - Precipitation of Driest Quarter; BIO18 - Precipitation of Warmest Quarter; BIO19 - Precipitation of Coldest Quarter. Variable BIO2 (Mean Diurnal Range) was excluded given its low biological significance, while BIO8 (Mean Temperature of Wettest Quarter) and BIO9 (Mean Temperature of Driest Quarter) were removed due to data artifacts. The bioclimatic variables were selected (bold and grey shade) by excluding highly correlated pairs (Pearson correlation > 0.7 or < -0.7 and VIF > 3), but also having into account their ecological meaning following previous ecological modelling studies for birds and herptiles in the Iberian Peninsula.

	BIO1	**BIO3**	**BIO4**	BIO5	BIO6	BIO7	**BIO10**	BIO11	BIO12	BIO13	BIO14	**BIO15**	**BIO16**	**BIO17**	BIO18	**BIO19**
BIO1	-	0.225	-0.175	0.715	0.785	-0.027	0.886	0.936	-0.479	-0.210	-0.328	0.227	-0.350	-0.566	-0.573	-0.227
**BIO3**	0.225	-	-0.290	0.203	0.183	0.023	0.102	0.297	-0.033	0.079	0.029	0.160	0.023	-0.196	-0.188	0.008
**BIO4**	-0.175	-0.290	-	0.496	-0.676	0.912	0.292	-0.504	-0.528	-0.535	-0.355	-0.387	-0.593	-0.214	-0.092	-0.611
BIO5	0.715	0.203	0.496	-	0.181	0.662	0.933	0.457	-0.778	-0.490	-0.570	0.029	-0.666	-0.717	-0.646	-0.612
BIO6	0.785	0.183	-0.676	0.181	-	-0.617	0.444	0.923	0.001	0.191	-0.008	0.412	0.131	-0.223	-0.272	0.155
BIO7	-0.027	0.023	0.912	0.662	-0.617	-	0.408	-0.338	-0.623	-0.538	-0.450	-0.291	-0.632	-0.403	-0.309	-0.608
**BIO10**	0.886	0.102	0.292	0.933	0.444	0.408	-	0.678	-0.714	-0.429	-0.497	0.074	-0.606	-0.670	-0.629	-0.511
**BIO11**	0.936	0.297	-0.504	0.457	0.923	-0.338	0.678	-	-0.241	0.015	-0.177	0.356	-0.097	-0.437	-0.493	0.011
BIO12	-0.479	-0.033	-0.528	-0.778	0.001	-0.623	-0.714	-0.241	-	0.805	0.738	0.184	0.925	0.821	0.716	0.788
BIO13	-0.210	0.079	-0.535	-0.490	0.191	-0.538	-0.429	0.015	0.805	-	0.457	0.656	0.908	0.480	0.345	0.594
BIO14	-0.328	0.029	-0.355	-0.570	-0.008	-0.450	-0.497	-0.177	0.738	0.457	-	-0.157	0.535	0.810	0.742	0.543
**BIO15**	0.227	0.160	-0.387	0.029	0.412	-0.291	0.074	0.356	0.184	0.656	-0.157	-	0.471	-0.196	-0.276	0.123
**BIO16**	-0.350	0.023	-0.593	-0.666	0.131	-0.632	-0.606	-0.097	0.925	0.908	0.535	0.471	-	0.633	0.516	0.688
**BIO17**	-0.566	-0.196	-0.214	-0.717	-0.223	-0.403	-0.670	-0.437	0.821	0.480	0.810	-0.196	0.633	-	0.931	0.490
BIO18	-0.573	-0.188	-0.092	-0.646	-0.272	-0.309	-0.629	-0.493	0.716	0.345	0.742	-0.276	0.516	0.931	-	0.324
**BIO19**	-0.227	0.008	-0.611	-0.612	0.155	-0.608	-0.511	0.011	0.788	0.594	0.543	0.123	0.688	0.490	0.324	-
